# Complement-dependent cytotoxicity of human autoantibodies against myelin oligodendrocyte glycoprotein

**DOI:** 10.3389/fnins.2023.1014071

**Published:** 2023-02-01

**Authors:** Kuniko Kohyama, Hiroya Nishida, Kimihiko Kaneko, Tatsuro Misu, Ichiro Nakashima, Hiroshi Sakuma

**Affiliations:** ^1^Department of Brain and Neurosciences, Tokyo Metropolitan Institute of Medical Science, Tokyo, Japan; ^2^Department of Neurology, Tohoku University Graduate School of Medicine, Sendai, Japan; ^3^Department of Neurology, Tohoku Medical and Pharmaceutical University, Sendai, Japan

**Keywords:** myelin oligodendrocyte glycoprotein, autoantibody, acquired demyelinating syndromes, cytotoxicity, complement

## Abstract

**Background:**

The autoantibody to myelin oligodendrocyte glycoprotein (MOG), a component of the central nervous system myelin, has been identified in a subset of demyelinating diseases. However, there is no convincing evidence to support the direct pathogenic contribution of this autoantibody.

**Objective:**

To elucidate the role of anti-MOG autoantibodies in human demyelinating disorders, we assessed the effect of autoantibodies on MOG-expressing cells.

**Methods:**

Mammalian cells expressing the human MOG protein reacted with human anti-MOG autoantibodies in the presence or absence of complement. Sera from 86 patients and 11 healthy sera were used. We analyzed anti-MOG antibody titers, IgG subclass, and their cytotoxic ability in sera from patients with various neurological diseases. Membrane attack complex (MAC) formation was examined by detection of complement C9 or C9neo with western blot or flow cytometry.

**Results:**

Among 86 patients, 40 were determined to be MOG-IgG-positive and 46 were negative. Anti-MOG-positive sera, but not -negative sera, caused cell death in MOG-expressing cells. This cytotoxic effect was disappeared after heat inactivation of sera. Importantly, anti-MOG IgG and externally added complement were necessary for sufficient cytotoxic effects. Anti-MOG autoantibodies were histologically colocalized with complement and formed a membrane attack complex consisting of anti-MOG IgG and complement factors.

**Conclusion:**

The human MOG antibody specifically killed MOG-expressing cells *in vitro* in the presence of externally added complement. Membrane attack complexes were formed on the cells, indicating that this autoantibody activated complement-mediated cytotoxicity. Further studies in larger numbers of patients are needed to characterize the role of complement in MOGAD.

## 1. Introduction

Acquired demyelinating syndrome (ADS) is a group of disorders characterized by demyelinating episodes of the central nervous system. Multiple sclerosis (MS) is one of the most common autoimmune diseases of the central nervous system and is believed to be mediated by autoreactive T cells that react to unknown myelin antigens. Neuromyelitis optica spectrum disorder (NMOSD), another autoimmune demyelinating disease, is caused by autoantibodies to the astrocytic water channel aquaporin-4 (AQP4). The discovery of anti-AQP4 antibodies highlighted the important role of autoantibodies in the pathogenesis of ADS.

Recently, autoantibodies to myelin oligodendrocyte glycoprotein (MOG), a component of the central nervous system myelin, were identified in a subset of demyelinating diseases (Reindl et al., 2013; Reindl and Waters, 2019). This autoantibody was initially identified in adult patients with MS (Lalive et al., 2006; Zhou et al., 2006) but is more commonly found in childhood acute disseminated encephalomyelitis (ADEM) (Brilot et al., 2009; Probstel et al., 2011). Further studies showed that this autoantibody was also associated with AQP4-antibody-negative NMOSD and cortical encephalitis (Mader et al., 2011; Ogawa et al., 2017). Despite its clinical heterogeneity, MOG antibody-associated disease (MOGAD) has been confirmed as a distinct demyelinating disease.

MOG is a minor component of CNS myelin, representing approximately 0.01–0.05% of the total myelin proteins (Amiguet et al., 1992). MOG is preferentially localized on the outermost surface of the myelin sheath and thus is directly exposed to autoantibodies in the extracellular milieu (Breithaupt et al., 2003). Immunization of C57BL/6 mice with the MOG peptide results in progressive paralysis, known as EAE, in which MOG-specific CD4^+^ T cells play a central role (Constantinescu et al., 2011). In addition, LEW.1AV1 rats immunized with MOG developed EAE with optic nerve and spinal cord involvement that mimicked neuromyelitis optica (Sakuma et al., 2004). Based on these observations, it has been speculated that MOG is a candidate autoantigen in human demyelinating disorders.

There has been some clinical evidence supporting the pathogenicity of this autoantibody. First, the clinical presentations observed in anti-MOG autoantibody-positive patients resemble those seen in experimental autoimmune encephalomyelitis (EAE) induced by MOG immunization (Reindl et al., 2013). Second, MOG autoantibodies have been reported to have the capacity to activate the complement cascade (Mader et al., 2011). Despite these clinical observations, there is no convincing evidence to support the direct pathogenic role of this autoantibody in human demyelinating diseases.

To address this issue, we studied the effect of human anti-MOG autoantibodies on mammalian cells expressing the human MOG protein on their surface. We demonstrated that anti-MOG autoantibodies cause cell death in MOG-expressing cells in a complement-dependent manner.

## 2. Materials and methods

### 2.1. Human samples

Human sera were obtained from 86 patients aged 0–61 years (72 children and 14 adults) who had ADS (MS, multiple sclerosis; EM, encephalomyelitis; ON, optic neuritis; NMO, neuromyelitis optica; etc.) from 41 hospitals. The patient details are shown in Supplementary Table 1. Sera were also collected from 11 healthy subjects (age range, 26–40 years).

### 2.2. Cells

A cell line expressing human MOG and green fluorescence protein (GFP) fusion protein (MOG-GFP cells) was generated as follows. First, the full-length human MOG α1 sequence (NM_206809.3) was cloned from a frozen block of human brain tissue obtained from the MS and Parkinson’s tissue bank (London). The GFP sequence was then fused to the C-terminal of MOG α1 with a short linker, and the resulting construct was ligated into pcDNA5/FRT/TO (Life Technologies Japan, Tokyo) (Supplementary Figure 1A). This MOG-GFP expressing vector and Flp-recombinase-producing pOG44 vector (Life Technologies Japan, Tokyo) were co-transfected into Flp-In 293 cells, then screened with hygromycin B. In parallel, to generate a control cell line (empty cells), intact pcDNA/FRT/TO and pOG44 were co-transfected into Flp-In 293 and screened as described above.

### 2.3. Titration of anti-MOG autoantibody

MOG-GFP cells and empty cells were mixed in a 1:1 ratio and prepared in microtubes to make aliquots (2 × 10^5^ cells/tube). After incubation with 10% fetal bovine serum, cell aliquots were reacted with diluted human serum (1:400) for 1 h. Then, they were incubated with Alexa fluor 647 labeled anti-human IgG (Jackson ImmunoResearch, PA, United States) for 15 min and analyzed using a BD FACSCanto II flow cytometer (BD Biosciences, CA, United States). In the experiment for IgG subclass assessment, phycoerythrin (PE)-labeled anti-human IgG 1, 2, 3, or 4 (Abcam Japan, Tokyo) was used as a second antibody. Because MOG-GFP cells and empty cells were separated by their green fluorescent intensity, the net value of anti-MOG reactivity was calculated from the difference in Alexa Fluor 647 or PE intensity between MOG-GFP cells and empty cells. To obtain the anti-MOG antibody titers, the net value was applied to the calibration curve drawn by the anti-MOG monoclonal antibody (8–18C5; Merck Millipore, Frankfurter) and following DyLight 649-labeled anti-mouse IgG (Jackson ImmunoResearch, PA, United States). The titers were then expressed as a ratio to a standard serum to correct for variation between experiments. Measurements were performed several times for each sample, and the mean titer ratios were calculated. The FlowJo software (BD Biosciences, CA, United States) was used for the analysis. Gating strategy was indicated in Supplementary Figure 2.

### 2.4. Cytotoxicity assay

We measured the cytotoxic activity of the patients’ samples of MOG-expressing cells. Anti-MOG-positive sera were added to the MOG-expressing cell aliquots (mixture of each 1 × 10^5^ cells of MOG-GFP and empty cells) at 20% v/v in DMEM and incubated at room temperature for 30 min, followed by sequential reaction with 20% v/v of Low-Tox-H rabbit complement (Cedarlane, Ontario) or pooled human complement serum (Innovative Research, MI, United States) at 37°C for 30 min. Cells were washed once, stained with propidium iodide (PI), and analyzed using a BD LSRFortessa flow cytometer (BD Biosciences, CA, United States). The rate of cells with damaged membranes representing cytotoxicity was calculated from the proportion of PI-positive MOG-GFP cells among all MOG-GFP cells. The FlowJo software was used for the analysis. Gating strategy was indicated in Supplementary Figure 2.

### 2.5. Cell viability assay (MTS assay)

To confirm the cytotoxicity of anti-MOG-positive sera, we also performed viability assay using 6 MOG-IgG-positive sera and four healthy sera. MOG-GFP cells and empty cells were cultured in 96-well plates at a density of 2 × 10^4^ cells/well. The culture medium was replaced with fresh medium containing 20% v/v human serum and incubated at 37°C in a CO_2_ incubator for 30 min, followed by a sequential reaction with 20% v/v of rabbit complement overnight. Then, CellTiter 96 AQueous One Solution Reagent (Promega, WI, United States) was added and incubated for 30–60 min. OD_490_ was measured using a multiplate leader ARVO X3 (PerkinElmer Japan, Yokohama) to calculate cell viability.

### 2.6. Separation of IgG and non-IgG fractions from human serum

To identify the factor(s) that determine the cytotoxic activity, we separated some human sera into IgG and non-IgG fractions. The IgG fraction was isolated from 50 μl of serum with Ab Spin Trap (GE Healthcare Japan, Tokyo) according to the manufacturer’s instructions and concentrated at 50 μl by using Amicon Ultra-0.5 (Merck Millipore, Frankfurter). Another 50 μl of serum was incubated with Protein G Sepharose Fast Flow (GE Healthcare Japan, Tokyo) overnight and centrifuged to collect the supernatant as a non-IgG fraction. The anti-MOG IgG activities of both fractions were measured using the same method as for the autoantibody titration.

### 2.7. Immunofluorescence

MOG-GFP cells or empty cells were cultured in Lab-Tek II 8-well chamber slides (Nunc, Roskilde). After fixation with 4% paraformaldehyde and blocking with Protein Block (DAKO Japan, Tokyo), they were reacted with human samples or anti-MOG antibody (8–18C5), followed by Alexa fluor 647 labeled anti-human IgG or DyLight 649 labeled anti-mouse IgG. In some experiments, cells that reacted with human sera and rabbit complement in the chambers were stained with Alexa fluor 647 labeled anti-human IgG and anti-complement C9 antibody (Santa Cruz Biotechnology, CA, United States), followed by Cy3 labeled anti-mouse IgG (Jackson ImmunoResearch, PA, United States) to visualize immune-complex formation. Images were obtained using a FLUOVIEW FV1000 confocal microscope (Olympus, Tokyo, Japan).

### 2.8. Western blot

To detect immune complexes, cells that reacted with human sera and rabbit complement were dissolved in NuPAGE LDS Sample Buffer (Life Technologies Japan, Tokyo) and loaded onto SDS-PAGE using NuPAGE 10% Bis-Tris protein gel (Life Technologies Japan, Tokyo). The separated proteins were transferred to polyvinylidene fluoride membranes and blocked with Block Ace (DS Pharma Biomedical, Osaka, Japan). The blots were then reacted with anti-complement C9 antibody and mouse TrueBlot ULTRA anti-mouse Ig HRP (Rockland, PA, United States). After reacting with ECL Select (GE Healthcare Japan, Tokyo), the bands were visualized using ImageQuant LAS 4000 (GE Healthcare Japan, Tokyo).

### 2.9. Analysis of MAC formation

Mixture of MOG-GFP cells and empty cells (1:1) were reacted with 20% v/v of human sera at room temperature for 30 min, followed by sequential reaction with 20% v/v of rabbit complement at 37°C for 30 min in the same way as cytotoxicity assay. After washing, cells were reacted with anti-C9neo antibody [C5b-9 antibody (aE11); Novus Biologicals, CO, United States] and subsequent DyLight 649 labeled anti-mouse IgG. C9neo fluorescence was quantified in each of MOG-expressing cells and empty cells using a BD FACSCanto II flow cytometer.

### 2.10. Statistical analysis

We used the software R for statistical analysis. To distinguish MOG-IgG-positive sera and -negative sera, ROC curve analysis was applied to validated patients’ sera and healthy sera. A non-parametric test (Kruskal–Wallis test) was done to estimate differences among multiple groups, such as differences in anti-MOG antibody titers among various diseases. Mann–Whitney *U* test was used in comparison between two groups, such as comparison of cytotoxicity between MOG-IgG-positive sera and -negative sera. Kruskal–Wallis test and Steel–Dwass test were used in comparison among three or more groups, such as comparison of anti-MOG antibody titer among various disease groups. The correlation between anti-MOG antibody titer and cytotoxicity to MOG-expressing cells was estimated from the Spearman’s rank correlation coefficient.

## 3. Results

### 3.1. MOG expression by MOG-GFP cells

The MOG-expressing vector was designed to express the fusion protein of human MOGα1 and GFP on the surface of transfected cells (Supplementary Figure 1A), and we confirmed that MOG and GFP were expressed using immunofluorescence (Supplementary Figure 1B). Anti-MOG monoclonal antibody (8–18C5) stained the surface of MOG-GFP cells, which were merged with GFP. Furthermore, the patient’s serum was positive for anti-MOG antibody (16–21S), but not healthy serum (OM33M), stained MOG-GFP cells. Empty cells did not react with 8–18C5 or the patient’s serum (data not shown).

### 3.2. Development of a method for autoantibody titration using flow cytometry

Using MOG-GFP cells, we developed a flow cytometric assay to quantify the titer of anti-MOG autoantibodies in human sera. Human sera positive for anti-MOG antibody, as well as an anti-MOG monoclonal antibody, had enough reactivity with MOG-GFP cells to be detected by flow cytometry (Supplementary Figure 3A). The mean fluorescence intensity (MFI) for antibody titers of the same samples was reproducible among examinations (data not shown). Moreover, repetitive tests of drawing a calibration curve with 8–18C5 showed high quantitativity (a representative graph is shown in Supplementary Figure 3B). Based on these observations, we validated the method of anti-MOG antibody titration using flow cytometry. According to the linearity of the titers obtained from the calibration curve, we determined the dilution factors of the samples as 400-fold dilution for serum. The measured antibody titers were expressed as arbitrary units relative to the 8–18C5 concentration.

### 3.3. The titer of anti-MOG autoantibody

**FIGURE 1:**
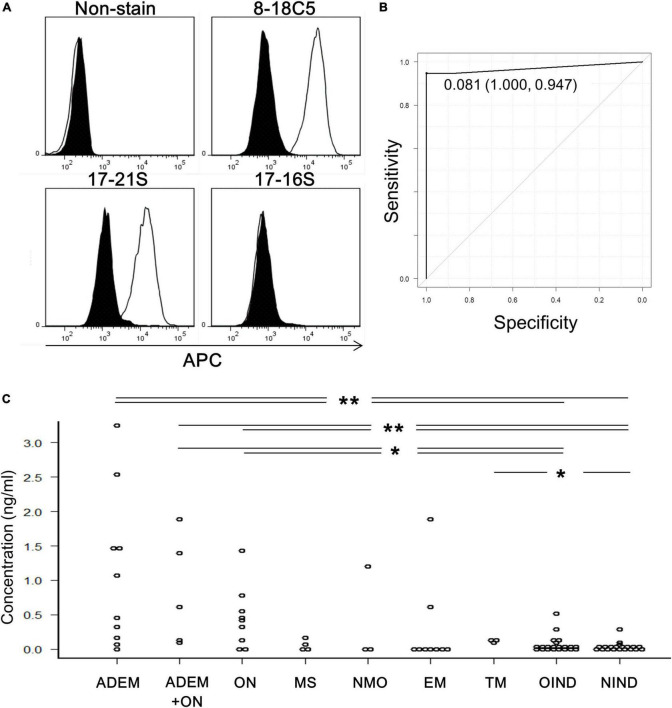
Titration of anti-MOG antibody by flow cytometry. **(A)** Representative histograms of cells stained with only the second antibody (non-stain), 8–18C5, or patients’ sera (17–21S; anti-MOG positive, 17–16S; anti-MOG negative). The APC intensity of empty cells (closed) and MOG-GFP cells (open) are shown. **(B)** An ROC curve from 35 samples for evaluation of the screening efficacy and for identification of a cutoff point of serum anti-MOG titer level. **(C)** Anti-MOG antibody titer ratios in various diseases (ADEM; 10, ADEM + ON; 5, ON; 9, MS; 4, NMO; 3, EM; 9, TM; 3, OIND; 21, and NIND; 20). Statistical analysis was performed using Kruskal–Wallis test and Steel–Dwass test (*P < 0.05, **P < 0.1).

Human anti-MOG-positive sera, as well as anti-MOG monoclonal antibody (8–18C5), had enough reactivity with MOG-GFP cells to be detected by flow cytometry (Figure 1A). Therefore, we measured the anti-MOG antibody titer ratio using flow cytometry in a large number of patient sera and several healthy samples (Supplementary Table 1). Firstly, to examine the validity of our assay, we compared our data with the results from Tohoku University with proven experience in established MOG antibody assays. All 18 of our positive sera tested positive, while only one of our ten negative sera was positive and the other nine were negative. We then used this data to determine the cut-off value for our assay. We included 19 validated sera plus 6 negative samples derived from healthy subjects. In total, 19 positive and 15 negative samples were used for ROC analysis, yielding a cut-off value of 0.081 and an area under the curve of 0.97 (Figure 1B). Based on this cut-off value, 40 patients were determined to be MOG-IgG-positive and 46 patients were MOG-IgG-negative. A comparison of antibody titers among sera from patients with various acquired demyelinating syndromes revealed that the groups of ADEM (range, 0–3.25; mean 1.08 ± 1.11) and ADEM + ON (ADEM (R); RDEM) (0.11–1.89; 0.83 ± 0.79) showed a high titer ratio and a part of the samples in the groups of ON (0–1.42; 0.46 ± 0.45), NMO (0–1.19; 0.40 ± 0.68), and EM (0–1.87; 0.28 ± 0.63) showed relatively high value. Meanwhile, the groups of MS (0–0.15; 0.05 ± 0.07), transverse myelitis (TM) (0.11–0.14; 0.12 ± 0.01), other inflammatory neurological disease (OIND) (0–0.51; 0.06 ± 0.13), and non-inflammatory neurological disease (NIND) (0–0.31; 0.02 ± 0.07) showed a low titer ratio. Importantly, no anti-MOG titer was detected in healthy samples (0–0). We found there were significant differences between the ADEM, ADEM + ON, and ON groups and the OIND and NIND groups and between TM group and NIND group. *P*-values were 0.004 (ADEM vs. OIND), 0.0007 (ADEM vs. NIND), 0.021 (ADEM + ON vs. OIND), 0.004 (ADEM + ON vs. NIND), 0.038 (ON vs. OIND), 0.008 (ON vs. NIND), and 0.038 (TM vs. NIND) (Figure 1C).

### 3.4. Complement-dependent cytotoxicity of anti-MOG autoantibody

**FIGURE 2:**
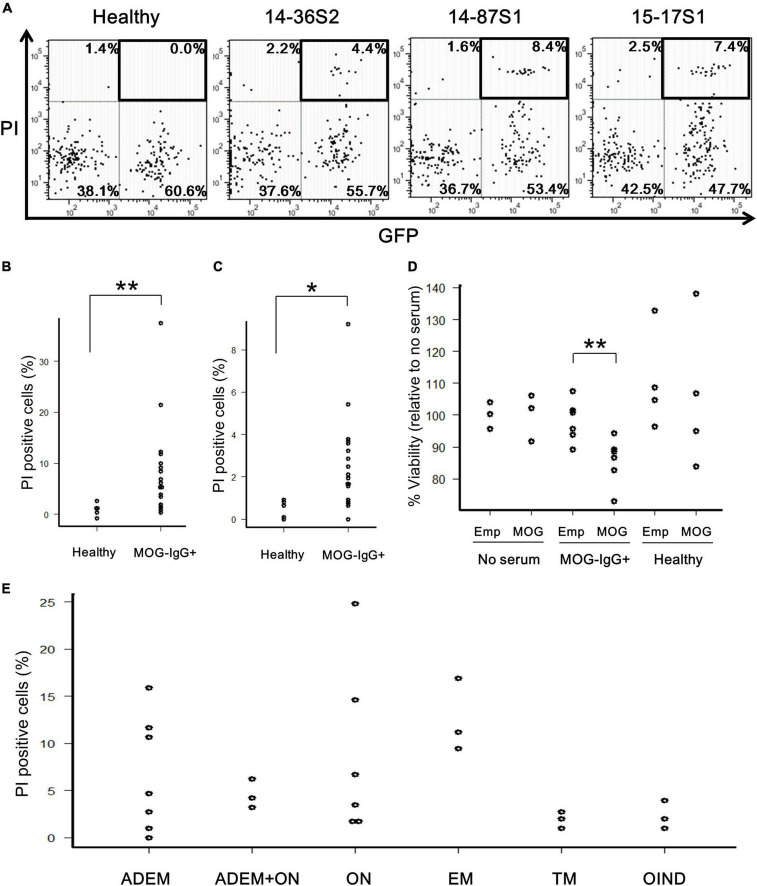
Cytotoxic effects of anti-MOG antibody in MOG-expressing cells. **(A)** Representative FACS plots of the cytotoxicity assay. The upper-right GFP^+^PI^+^ populations (boxed) in the dot plots show damaged MOG-GFP cells. Each proportion of PI-positive cells is calculated as: [number of GFP^+^PI^+^ (or GFP^–^PI^+^) cells]/[total number of MOG-GFP (or empty) cells]. Cell number in viable gate was considerably reduced because lots of cells died by complement assay. **(B,C)** Proportion of PI-positive cells among total GFP^+^ cells in the healthy (*n* = 6) or anti-MOG-positive (*n* = 18) groups. Panel **(B)** shows a result with rabbit complement and panel **(C)** shows one with human complement. Statistical significance was determined by Mann–Whitney *U* test (*P < 0.05, **P < 0.01). **(D)** Cell viability of empty (Emp) and MOG-GFP cells (MOG) treated with/without sera measured by the MTS assay. The sample size was no serum (*n* = 3), anti-MOG positive serum (*n* = 6), and healthy serum (*n* = 4). Mann–Whitney *U* test was used for statistical comparison between Emp and MOG (**P < 0.01). **(E)** Main data tested for PI-positive cells are plotted by disease group (*n* ≥ 3 in each group): 27 patients with confirmed MOG antibody positivity, including ADEM (*n* = 7), ADEM + ON (*n* = 3), ON (*n* = 6), EM (*n* = 3), TM (*n* = 3), and OIND (*n* = 3). Statistical analysis was performed using Kruskal–Wallis test and Steel–Dwass test, and no differences were detected.

To determine whether the anti-MOG autoantibody is cytotoxic to MOG-expressing cells by binding to cell-surface antigens, we performed two types of *in vitro* assays. First, MOG-GFP cells were incubated with anti-MOG-positive sera in the presence of complement (rabbit or human) and analyzed by flow cytometry. Several anti-MOG-positive sera induced a large proportion of PI-positive (damaged) cells, specifically in MOG-GFP cells (Figure 2A). The net degree of damage of the cells induced by anti-MOG-positive sera was calculated by subtracting the PI-positive proportion of empty cells from that of MOG-GFP cells. The mean percentage of net PI-positive cells was significantly higher in the anti-MOG-positive sera than in the healthy sera (*P* = 0.007 for the test with rabbit complement; Figure 2B and *P* = 0.011 for the test with human complement; Figure 2C). Second, as a result of the MTS assay, the viability of MOG-GFP cells was significantly reduced after reaction with anti-MOG-positive sera and following rabbit complement, while that of empty cells was not affected by anti-MOG-positive sera (*P* = 0.009 for empty cells vs. MOG-GFP cells in anti-MOG-positive sera) (Figure 2D). Healthy sera did not change the viability of MOG-GFP or empty cells. These results demonstrate that anti-MOG-positive sera specifically have a cytotoxic effect on MOG-GFP cells in the presence of complement. No significant differences in cytotoxic efficiency were detected among sera from various disease groups (Figure 2E).

### 3.5. Correlation between anti-MOG antibody titers and cytotoxicity to MOG-GFP cells

**FIGURE 3:**
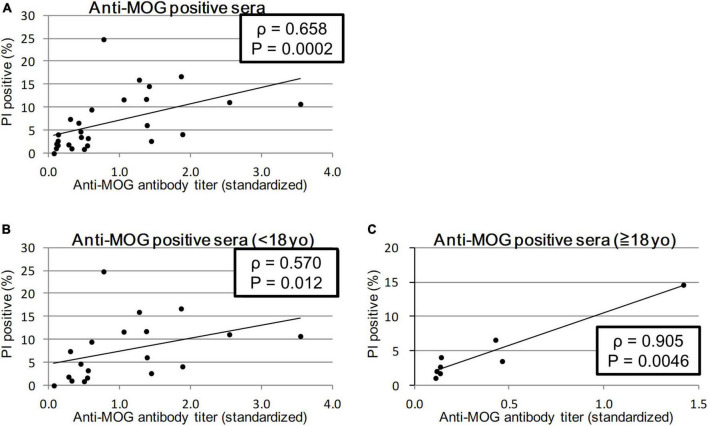
Correlation between anti-MOG antibody titer ratio and cytotoxicity (proportion of PI-positive cells). Twenty-seven MOG-IgG-positive samples including 19 from children and 8 from adults were examined. Among all patients (sera with the highest antibody titer of each patient) **(A)** and comparison of child patients (<18 years old) **(B)**, and adult patients (≥18 years old) **(C)**. Antibody titers are adjusted for the value of standard serum for each experiment. Spearman’s rank correlation coefficient (ρ) and *P*-values are calculated and shown in each graph.

We calculated the Spearman’s rank correlation coefficient between the anti-MOG antibody titer and MOG-GFP cell cytotoxicity indicated by PI staining. Sera from all patients showed a significant moderate correlation between antibody titer and cytotoxicity (ρ = 0.658, *P* = 0.0003; Figure 3A). When sera from the child group (<18 years) and the adult group (≥18 years) were analyzed separately, while the former had a moderate correlation (ρ = 0.570, *P* = 0.012), the latter had a close correlation (ρ = 0.905, *P* = 0.0046) (Figures 3B, C).

### 3.6. Anti-MOG IgG and externally added complement are necessary for a sufficient cytotoxic effect

**FIGURE 4:**
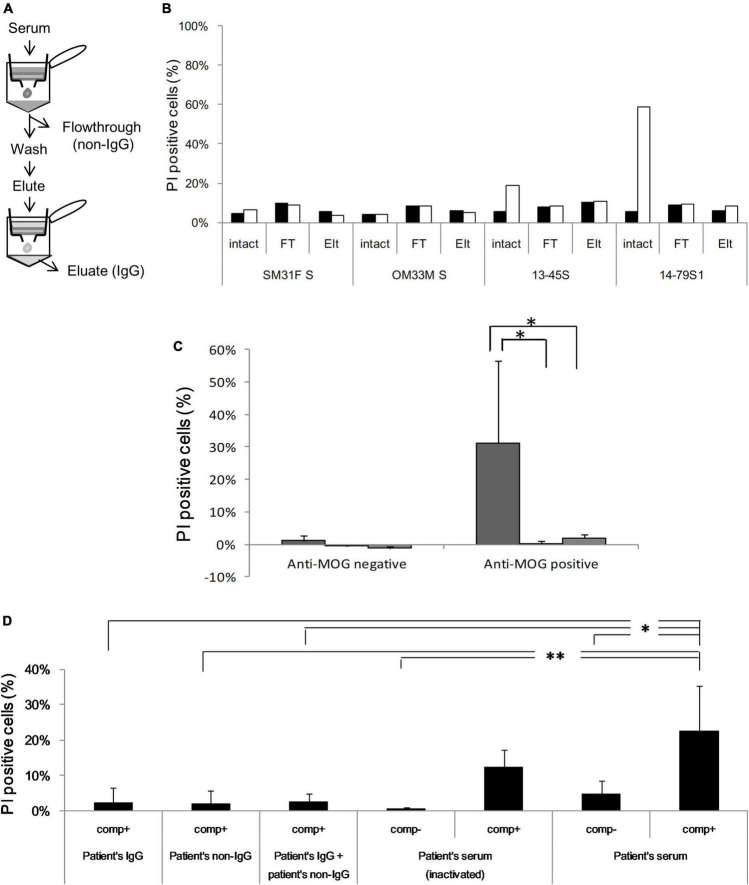
**(A)** Serum is fractionated into IgG [eluate (Elt)] and non-IgG [flowthrough (FT)] using protein G, and anti-MOG antibody activity is assessed in each fraction by flow cytometry. **(B)** Cytotoxicity against MOG-GFP cells is analyzed in each fraction of healthy sera (SM31F S and OM33M S) and patients’ sera (13–45S, 14–79S1, 14–79S2, and 14–87S). Closed bar; empty cell, open bar; MOG-GFP cells. **(C)** Cytotoxicity in each fraction of anti-MOG-negative (*n* = 2) or anti-MOG-positive (*n* = 4) samples. The statistical difference of each fraction to total serum was determined by Student’s *t*-test (*P < 0.05). Left bar; total serum, middle bar; flowthrough, right bar; eluate. **(D)** Various combinations of patients’ serum fractions and rabbit complement were tested by cytotoxicity assay. The same examinations are performed three times, and the mean data is shown. Statistical analysis was performed using Tukey–Kramer test (*P < 0.05, ^**^P < 0.01). “Comp” in the figure is the abbreviation of complement.

To identify the necessary factors for cytotoxic effects, we isolated the protein G-binding fraction (IgG) and protein G-unbinding fraction (non-IgG) from several sera. First, we examined their ability to bind to MOG by flow cytometry and confirmed that the IgG fraction had a similar MOG-binding ability as total serum, while the non-IgG fraction had no MOG-binding ability, which indicated successful fractionation (Figure 4A and Supplementary Figure 4). However, both fractions had far lower cytotoxicity than total serum in the *in vitro* assay (*P* < 0.05; Figures 4B, C). To examine whether another factor is responsible for cytotoxicity in addition to IgG, we tested various combinations of fractions (IgG and non-IgG) from patients’ sera and heat-inactivated patients’ sera using a cytotoxicity assay (Figure 4D). In the absence of additional rabbit complement, PI-positive cells in both reactions with heat-inactivated serum and with intact serum were significantly lower than those with intact serum in the presence of additional complement (*P* < 0.01 and *P* < 0.05). Furthermore, in the presence of rabbit complement, all fractionated samples, including the combination of IgG and non-IgG fractions, damaged cells to a lower degree than the intact serum (*P* < 0.01 for the non-IgG fraction and *P* < 0.05 for the others).

### 3.7. Anti-MOG IgG forms a membrane attack complex

Human IgG and complement component 9 (C9), the final factor that participates in the classical complement pathway, were stained in MOG-GFP cells with both patient’s serum and rabbit complement and observed by immunofluorescence (IF) to examine the formation of the membrane attack complex. Both IgG and C9 were detected on the MOG-GFP cell surface when reacted with anti-MOG antiserum but not with healthy serum (Supplementary Figure 5A). Furthermore, when cell precipitation after reacting with serum and rabbit complement was loaded on SDS-PAGE and analyzed by western blotting, C9 was immunoprecipitated only when MOG-GFP cells were reacted with anti-MOG antiserum (Supplementary Figure 5B). Moreover, we analyzed C9neo binding to MOG-GFP cells by flow cytometry after incubation with human serum and rabbit complement. The C9neo binding degree of the MOG-IgG-positive group was significantly much higher than that of the healthy group (Supplementary Figure 6). These results suggested that a membrane attack complex consisting of anti-MOG IgG and complement factors was formed on the surface of MOG-GFP cells.

### 3.8. Anti-MOG IgG is mostly IgG1

Finally, we analyzed IgG subclasses of anti-MOG antibodies. Of the 12 sera (from 12 patients in the acute phase positive for anti-MOG antibody), 11 sera were exclusively IgG1, and one included IgG1 and IgG3. We did not detect IgG2 or IgG4 in these samples (Supplementary Table 2).

## 4. Discussion

Several studies have suggested the pathogenic potential of human anti-MOG autoantibodies. First, anti-MOG autoantibodies react exclusively with native MOG proteins that are expressed on the cell surface (Brilot et al., 2009). It has become increasingly clear that autoantibodies that bind to cell surface determinants of membrane-associated proteins are likely to be pathogenic (Vincent et al., 2011). Second, antibodies to MOG are predominantly of the IgG1 subtype and can mediate complement-dependent cytotoxicity (Mader et al., 2011; Waters et al., 2015). Third, longitudinal analysis of serum anti-MOG IgG indicated an association of a favorable clinical outcome in ADEM with a decrease in antibody titers over time (Di Pauli et al., 2011; Lopez-Chiriboga et al., 2018). Pathologically, perivenous inflammatory demyelination with loss of MOG-dominant myelin was a characteristic finding of MOG antibody-associated disease (Takai et al., 2020). Cultured oligodendrocytes incubated with purified IgG from MOG antibody-positive patients showed a loss of cytoskeleton organization (Dale et al., 2014). EAE induced in macaques by recombinant human MOG presented brain lesions comparable to human MOG autoantibody-associated demyelinating diseases (Serguera et al., 2019). The most straightforward way to show the pathogenicity of autoantibodies is to administer them to mice to test whether they can cause clinical or pathological changes that replicate human diseases. However, this strategy is not helpful in studying anti-MOG antibodies because the human anti-MOG antibody does not cross-react with murine MOG (Mayer et al., 2013).

To overcome this problem, we used human MOG-expressing cells that were also used to measure antibody titers. This method was useful to demonstrate the pathogenicity of the anti-AQP4 antibody (Hinson et al., 2007). We successfully demonstrated that the antibodies had cytotoxic effects on MOG-expressing cells but not on control cells in the presence of complement. Human anti-MOG antibodies have been reported to induce natural killer cell-mediated killing of MOG-expressing cells *in vitro* (Brilot et al., 2009). However, there have been no reports that humoral factors are sufficient to injure MOG-expressing cells.

To further support the pathogenicity of the anti-MOG antibody, we demonstrated a significant correlation between antibody titer and cytotoxicity. The correlation was more evident when sera from one patient were used. In other words, the cytotoxicity of autoantibodies differs from patient to patient and depends not only on antibody titers. It has been reported that there are at least seven different epitope patterns recognized by human anti-MOG antibodies, while longitudinal analysis of the same patient indicated constant epitope recognition (Mayer et al., 2013). Therefore, epitope patterns may be one of the determinants of the cytotoxicity of autoantibodies.

Complements play an important role in antibody-mediated autoimmune diseases. NMOSD is characterized by complement-dependent and independent AQP4-antibody-mediated astrocytopathies (Nishiyama et al., 2016). MOGAD pathology was also characterized by complement deposition in the absence of selective MOG protein loss (Hoftberger et al., 2020). However, perivascular deposits of activated complement in MOGAD were much less prominent than those of AQP4 antibody-positive NMOSD (Takai et al., 2020). This observation is supported by the finding that most anti-MOG antibodies require bivalent binding, leading to poor C1q binding (Macrini et al., 2021). Clinically, proteins indicative of systemic classical and alternative complement activation were substantially increased in patients with MOGAD (Keller et al., 2021). We showed that antibody-mediated cell damage depended on complement because heat-inactivated sera lost cytotoxic activity. Immunoprecipitation confirmed that autoantibodies were colocalized with complement. The addition of rabbit complement to sera significantly augmented cytotoxic activity. Although human sera also contain complement, the addition of sera from healthy or diseased subjects to heat-inactivated sera did not restore cytotoxicity. We speculate that the amount of complement contained in the sera was too small to fully activate the complement cascade. Therefore, although anti-MOG antibodies have cytotoxic activity, they may not be able to cause massive oligodendrocyte death unless the complement cascade is sufficiently activated. This is consistent with the clinical observation that MOG autoantibody-associated demyelinating diseases mostly had a substantial recovery (Armangue et al., 2020).

When a cytotoxicity assay was performed using human complement, the results were similar to those obtained when rabbit complement was used, but the PI-positive cell rate was rather low. We confirmed that complement inhibitors CD46 and CD59 were highly expressed on MOG-expressing cells by flow cytometry (data not shown), suggesting that they may suppress the cytotoxic response. Nishiyama et al. (2016) showed that under the same reaction conditions, xenogenic rabbit complement induced stronger cytotoxicity than allogenic human complement, and the difference disappeared when silencing inhibitory molecules such as CD55 and CD59 with siRNA. These results suggested that the human complement is as effective as rabbit complement in inducing cytotoxicity. The results in the present study may have also followed these findings.

Although MOG autoantibody-associated demyelinating diseases generally have a good prognosis, some cases show frequent relapses and residual symptoms (Ramanathan et al., 2018). Our data suggest that targeting humoral immunity is a promising therapeutic strategy for demyelinating disorders associated with anti-MOG antibodies. However, immunotherapies targeting B-cell or autoantibody for MOGAD have led to conflicting results. There have been reports of the efficacy of plasma exchange and intravenous immunoglobulin in relapsing demyelinating encephalomyelitis (Whittam et al., 2020a,b). Interestingly, although rituximab reduced relapse rates in MOGAD, many patients continued to relapse despite apparent B-cell depletion (Whittam et al., 2020a). Rituximab was less effective in MOGAD than in AQP4-IgG positive NMOSD (Durozard et al., 2020). In addition to these treatments, regulation of the complement system may be another therapeutic option for intractable cases.

The present study has several limitations. Although we used 8-18-C5 mouse monoclonal antibody as a standard to determine MOG antibody titers, the use of humanized MOG antibody will provide more reliable results. In the flow cytometric analysis on the cytotoxic effect of MOG antibodies, the number of gated cells was low because a lot of cells died during incubation with complement. Therefore, the test does not really reflect the entire effect of complement on MOG expressing cells. The small sample size is the shortage of this study and further studies in larger numbers of patients are needed to characterize the role of complement in MOGAD.

## Data availability statement

The original contributions presented in this study are included in this article/Supplementary material, further inquiries can be directed to the corresponding author.

## Ethics statement

The studies involving human participants were reviewed and approved by Institutional Review Board of the Tokyo Metropolitan Institute of Medical Science. Written informed consent to participate in this study was provided by the participants’ legal guardian/next of kin.

## Author contributions

KKo and HS contributed to the study conceptualization and drafted the manuscript. KKo contributed to the acquisition and analysis of the data. HN, KKa, TM, IN, and HS critically reviewed the manuscript and supervised the conduct of this study. All authors approved the final version of the manuscript for submission.

## Supplementary material

Supplementary Figure 1 Generation of MOG-GFP-expressing cells. **(A)** Design of the MOG-GFP expression vector. FRT, Flp recombination target site; Hygro, hygromycin resistance gene; P_CMV_, human cytomegalovirus immediate early promoter. The MOG-GFP fusion gene is inserted into multiple cloning sites in the promoter region. Once the vector is transfected into Flp-In cells together with pOG44, the cell obtains hygromycin resistance and expresses the MOG-GFP protein. **(B)** MOG expression confirmed by immunofluorescence analysis. Cells re incubated with commercial anti-MOG monoclonal antibody (8–18C5), anti-MOG antibody-positive serum (16–21S), or healthy serum (OM33M) and visualized with Cy3-labeled secondary antibody. Green, GFP; red, anti-MOG antibody; blue, DAPI. The scale bar indicates 20 μm.

Supplementary Figure 2 Gating strategy for flow cytometry. **(A–E)** The cell-based anti-MOG antibody quantification was performed using BD FACSCanto II flow cytometer. Viable Flp-In 293 singlet cell population was gated in FSC-A vs. SSC-A **(A)**, FSC-W vs. FSC-H **(B)**, and SSC-W vs. SSC-H plots **(C)** and separated to MOG-GFP cells and empty cells by fluorescent level **(D)**. Each cell population was calculated for the reactivity with human sera **(E)**. **(F–J)** The cytotoxicity level of anti-MOG sera was measured using BD LSRFortessa flow cytometer. Viable Flp-In 293 singlet cell population was gated in FSC-A vs. SSC-A **(F)**, FSC-W vs. FSC-H **(G)**, and SSC-W vs. SSC-H plots **(H)**. Then gated cells were divided into a quadrant; GFP+PI+, GFP+PI−, GFP−PI+, and GFP−PI− population **(I,J)**. Cell number in viable gate was considerably reduced because lots of cells died by complement assay.

Supplementary Figure 3 Detection of anti-MOG activity by flow cytometry. **(A)** After reaction with human sera (or monoclonal antibody) and following APC-labeled second antibody, a mixture of MOG-GFP (open) and empty cells (closed) are gated by green fluorescence and measured for anti-MOG antibody activity with mean fluorescence intensity (MFI) of APC. **(B)** A standard curve is drawn using 8–18C5 in each experiment.

Supplementary Figure 4 Serum was fractionated to IgG [eluate (Elt)] and non-IgG [flowthrough (FT)] using protein G as shown in Figure 4A. Anti-MOG antibody activity was assessed in each fraction by flow cytometry. Intact and Elt fractions of identical serum showed almost the same levels of anti-MOG activity, and the FT fractions did not show anti-MOG activity.

Supplementary Figure 5 Demonstration of membrane attack complex formation. **(A)** MOG-GFP cells react with patients’/healthy serum and rabbit complement and detected by immunofluorescence with a combination of anti-human IgG antibody and anti-complement C9 antibody. Green, GFP; blue, complement C9; red, human IgG. The scale bar indicates 20 μm. **(B)** Western blotting. MOG-GFP cells reacted with patients’/healthy serum and rabbit complement are loaded into SDS-PAGE and blotted protein is detected with anti-complement C9 antibody. The arrow shows complement C9 (ca. 90 kDa), and the dashed arrow shows probable cleaved complement C9 (ca. 45 kDa).

Supplementary Figure 6 Demonstration of membrane attack complex formation by flow cytometry. After the reaction with human sera and the rabbit complement same as cytotoxicity assay, the mixture of MOG-GFP and empty cells is stained with anti-C9neo antibody and detected by flow cytometer. **(A,B)** Representative histograms of the cells reacted to healthy **(A)** or MOG-IgG+ **(B)** serum. **(C)** Fluorescent level of C9neo was compared between the healthy group and the MOG-IgG positive group. Mann–Whitney *U* test was used for statistical comparison (^**^*P* < 0.01).

Supplementary Table 1 Profile of the patients included in the study. ADEM, acute disseminated encephalomyelitis; ON, optic neuritis; MS, multiple sclerosis; NMO, neuromyelitis optica; EM, encephalomyelitis; TM, transverse myelitis; OIND, other inflammatory neurological disease; NIND, non-inflammatory neurological disease.

Supplementary Table 2 IgG subclasses of anti-MOG antibodies. MOG-GFP cells are sequentially reacted with sera and IgG subclass-specific secondary antibodies and are analyzed by flow cytometry. The number of patients who are positive for each IgG subclass is shown. ADEM, acute disseminated encephalomyelitis; ON, optic neuritis; NMO, neuromyelitis optica; EM, encephalomyelitis.
