# Supplementary figures and images for: Complement-dependent cytotoxicity of human autoantibodies against myelin oligodendrocyte glycoprotein
Source: Front Neurosci. 2023 Feb 1;17:1014071. doi: 10.3389/fnins.2023.1014071 (PMC9930155; doi:10.3389/fnins.2023.1014071)

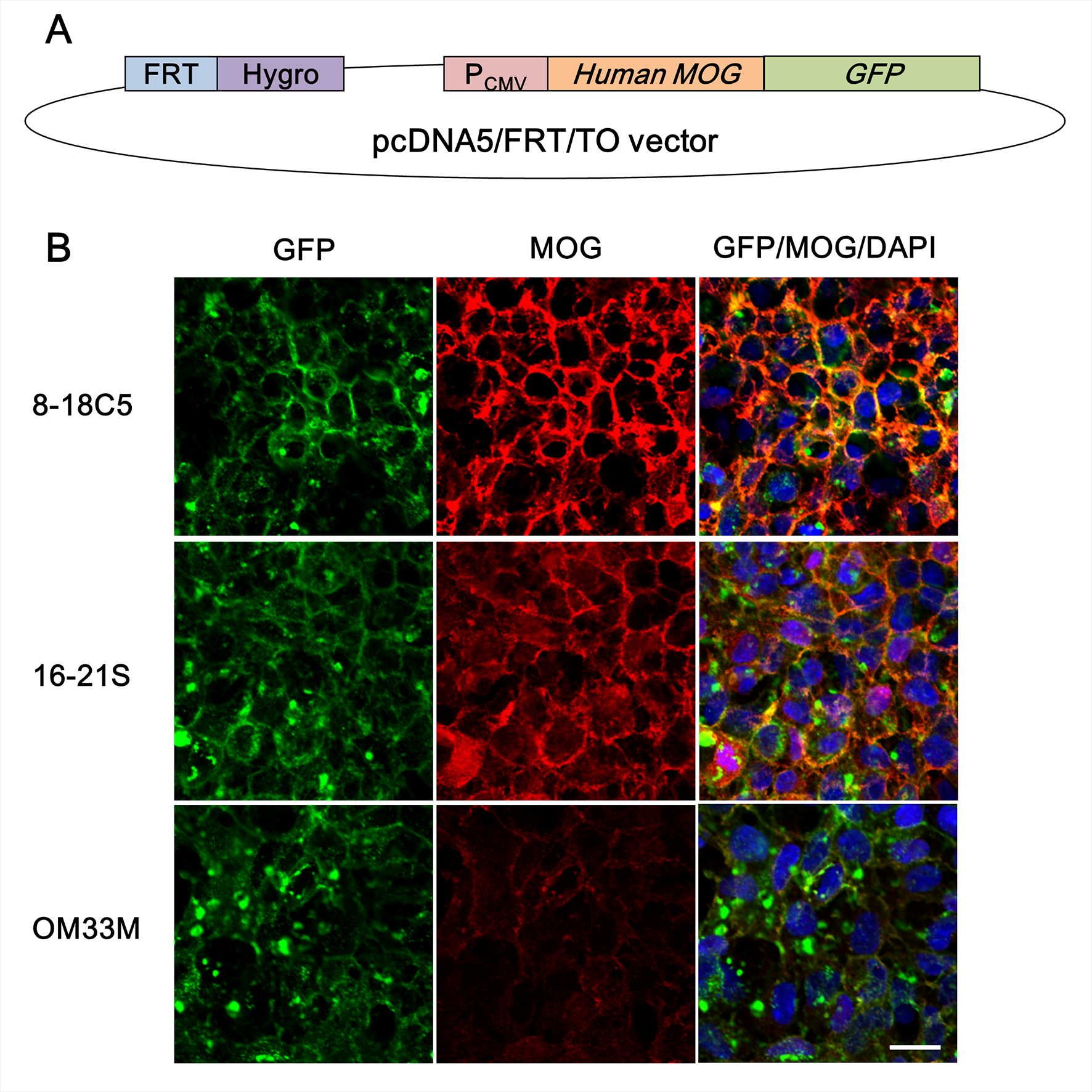

Supplement: Supplementary Figure 1 — Generation of MOG-GFP-expressing cells. (A) Design of the MOG-GFP expression vector. FRT, Flp recombination target site; Hygro, hygromycin resistance gene; PCMV, human cytomegalovirus immediate early promoter. The MOG-GFP fusion gene is inserted into multiple cloning sites in the promoter region. Once the vector is transfected into Flp-In cells together with pOG44, the cell obtains hygromycin resistance and expresses the MOG-GFP protein. (B) MOG expression confirmed by immunofluorescence analysis. Cells re incubated with commercial anti-MOG monoclonal antibody (8–18C5), anti-MOG antibody-positive serum (16–21S), or healthy serum (OM33M) and visualized with Cy3-labeled secondary antibody. Green, GFP; red, anti-MOG antibody; blue, DAPI. The scale bar indicates 20 μm. [file Image_1.TIF]

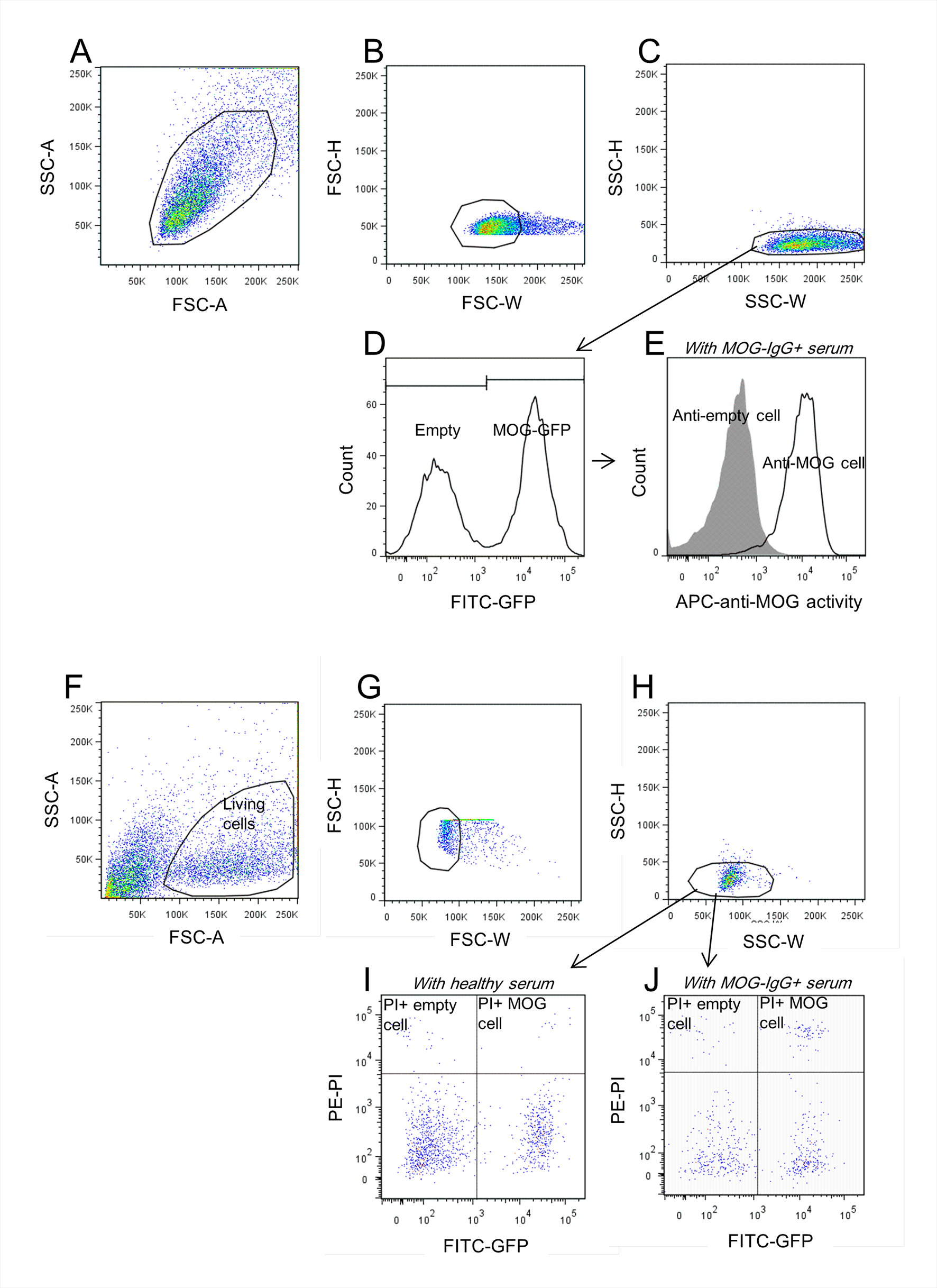

Supplement: Supplementary Figure 2 — Gating strategy for flow cytometry. (A–E) The cell-based anti-MOG antibody quantification was performed using BD FACSCanto II flow cytometer. Viable Flp-In 293 singlet cell population was gated in FSC-A vs. SSC-A (A), FSC-W vs. FSC-H (B), and SSC-W vs. SSC-H plots (C) and separated to MOG-GFP cells and empty cells by fluorescent level (D). Each cell population was calculated for the reactivity with human sera (E). (F–J) The cytotoxicity level of anti-MOG sera was measured using BD LSRFortessa flow cytometer. Viable Flp-In 293 singlet cell population was gated in FSC-A vs. SSC-A (F), FSC-W vs. FSC-H (G), and SSC-W vs. SSC-H plots (H). Then gated cells were divided into a quadrant; GFP+PI+, GFP+PI−, GFP−PI+, and GFP−PI− population (I,J). Cell number in viable gate was considerably reduced because lots of cells died by complement assay. [file Image_2.TIF]

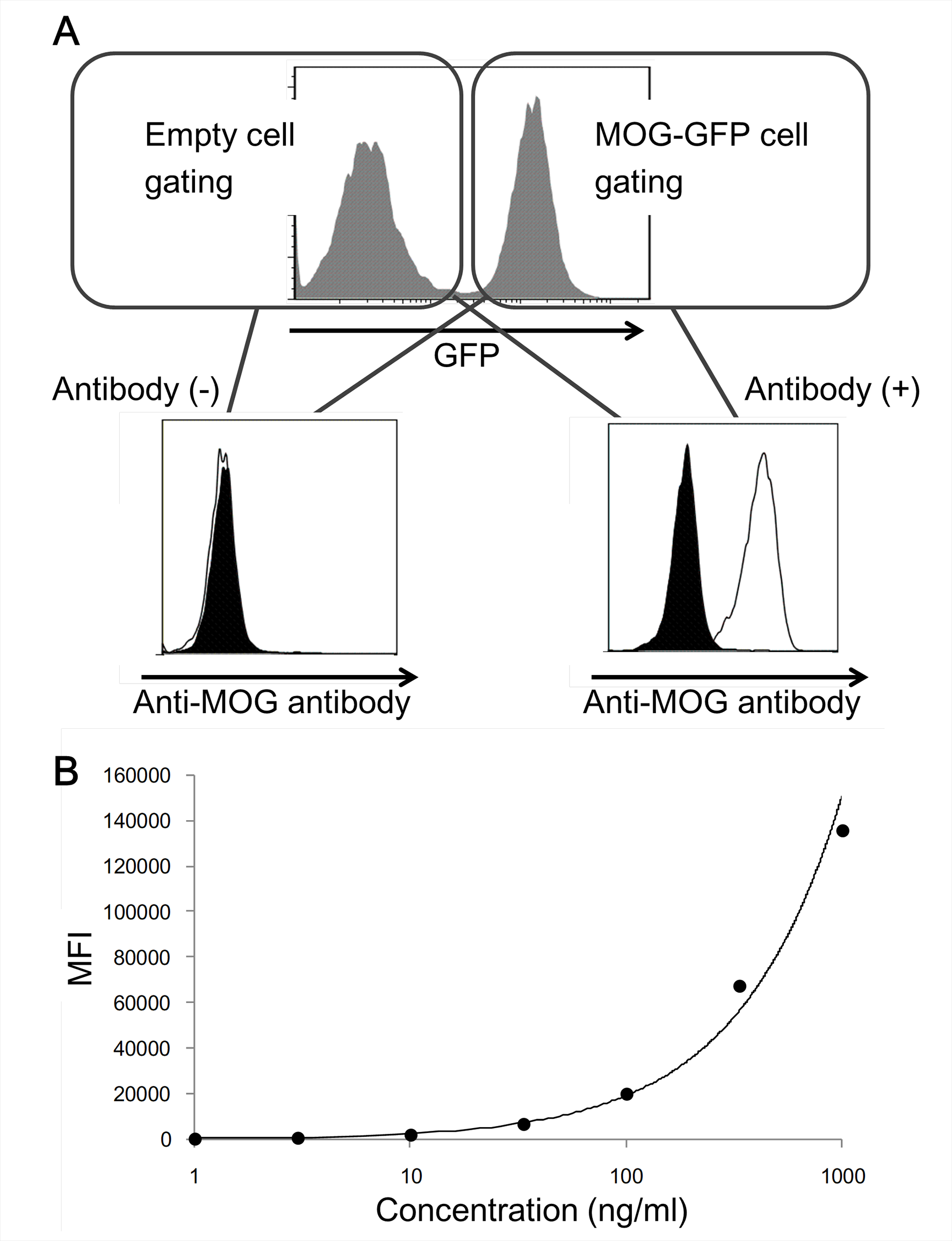

Supplement: Supplementary Figure 3 — Detection of anti-MOG activity by flow cytometry. (A) After reaction with human sera (or monoclonal antibody) and following APC-labeled second antibody, a mixture of MOG-GFP (open) and empty cells (closed) are gated by green fluorescence and measured for anti-MOG antibody activity with mean fluorescence intensity (MFI) of APC. (B) A standard curve is drawn using 8–18C5 in each experiment. [file Image_3.TIF]

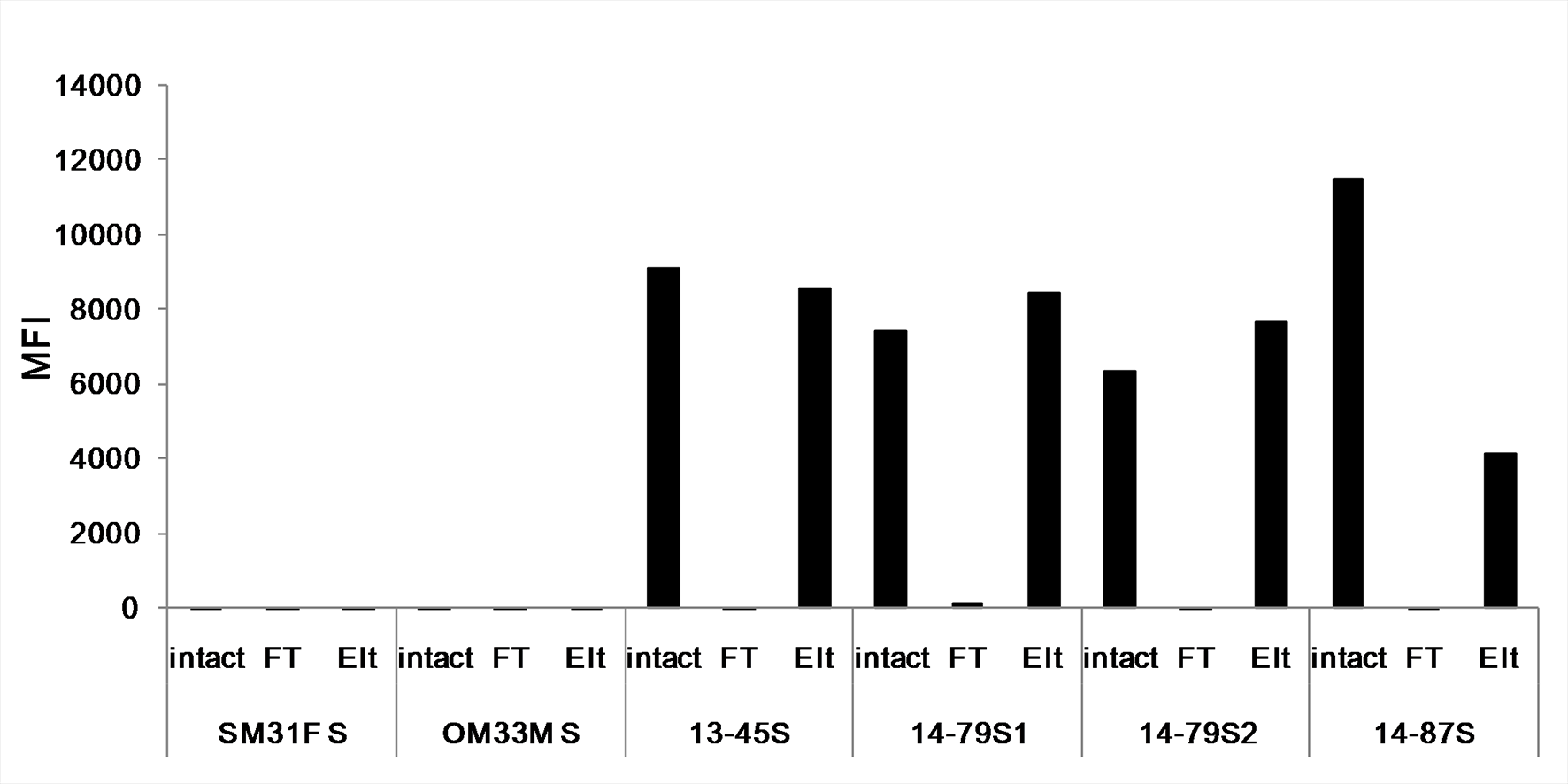

Supplement: Supplementary Figure 4 — Serum was fractionated to IgG [eluate (Elt)] and non-IgG [flowthrough (FT)] using protein G as shown in Figure 4A. Anti-MOG antibody activity was assessed in each fraction by flow cytometry. Intact and Elt fractions of identical serum showed almost the same levels of anti-MOG activity, and the FT fractions did not show anti-MOG activity. [file Image_4.TIF]

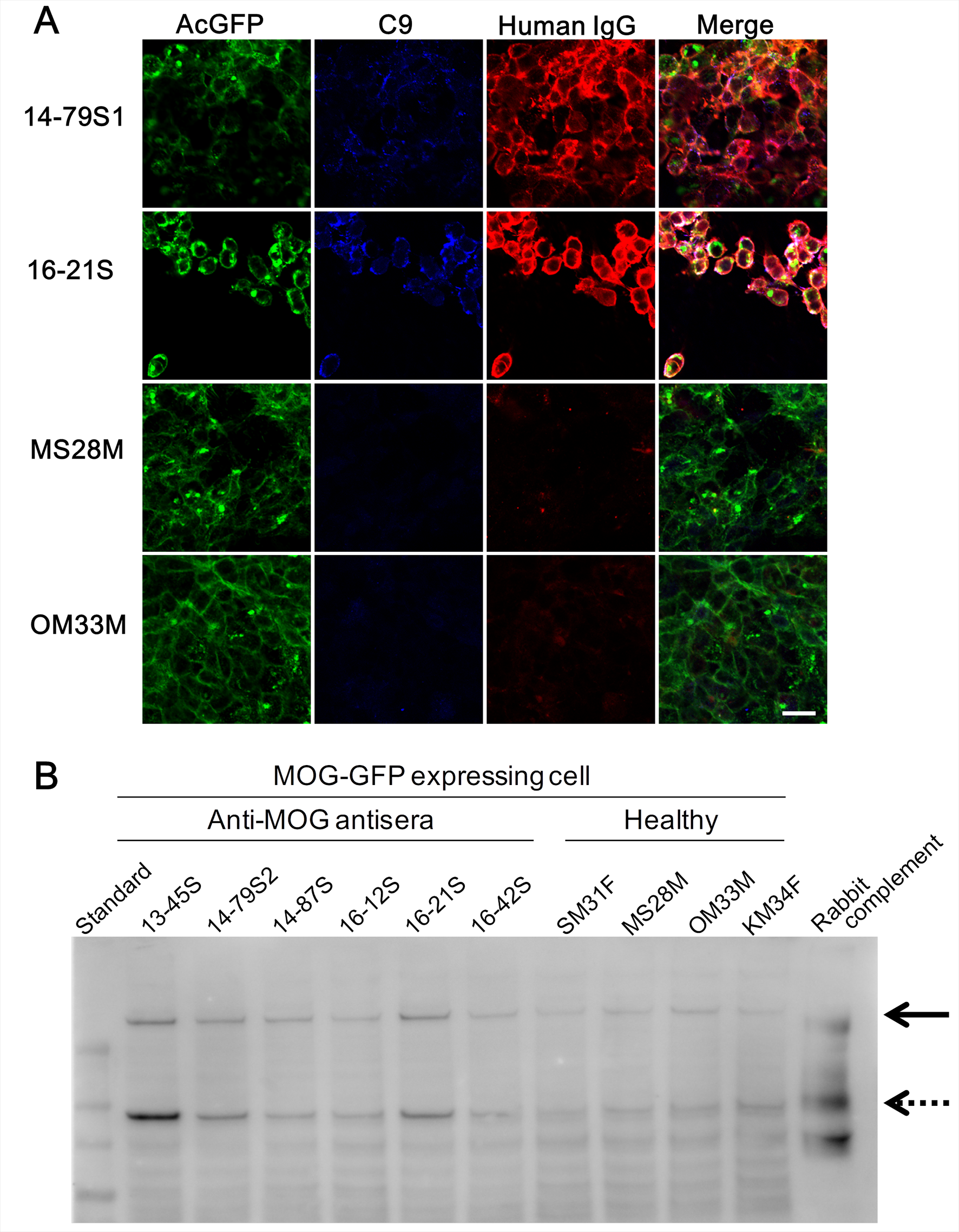

Supplement: Supplementary Figure 5 — Demonstration of membrane attack complex formation. (A) MOG-GFP cells react with patients’/healthy serum and rabbit complement and detected by immunofluorescence with a combination of anti-human IgG antibody and anti-complement C9 antibody. Green, GFP; blue, complement C9; red, human IgG. The scale bar indicates 20 μm. (B) Western blotting. MOG-GFP cells reacted with patients’/healthy serum and rabbit complement are loaded into SDS-PAGE and blotted protein is detected with anti-complement C9 antibody. The arrow shows complement C9 (ca. 90 kDa), and the dashed arrow shows probable cleaved complement C9 (ca. 45 kDa). [file Image_5.TIF]

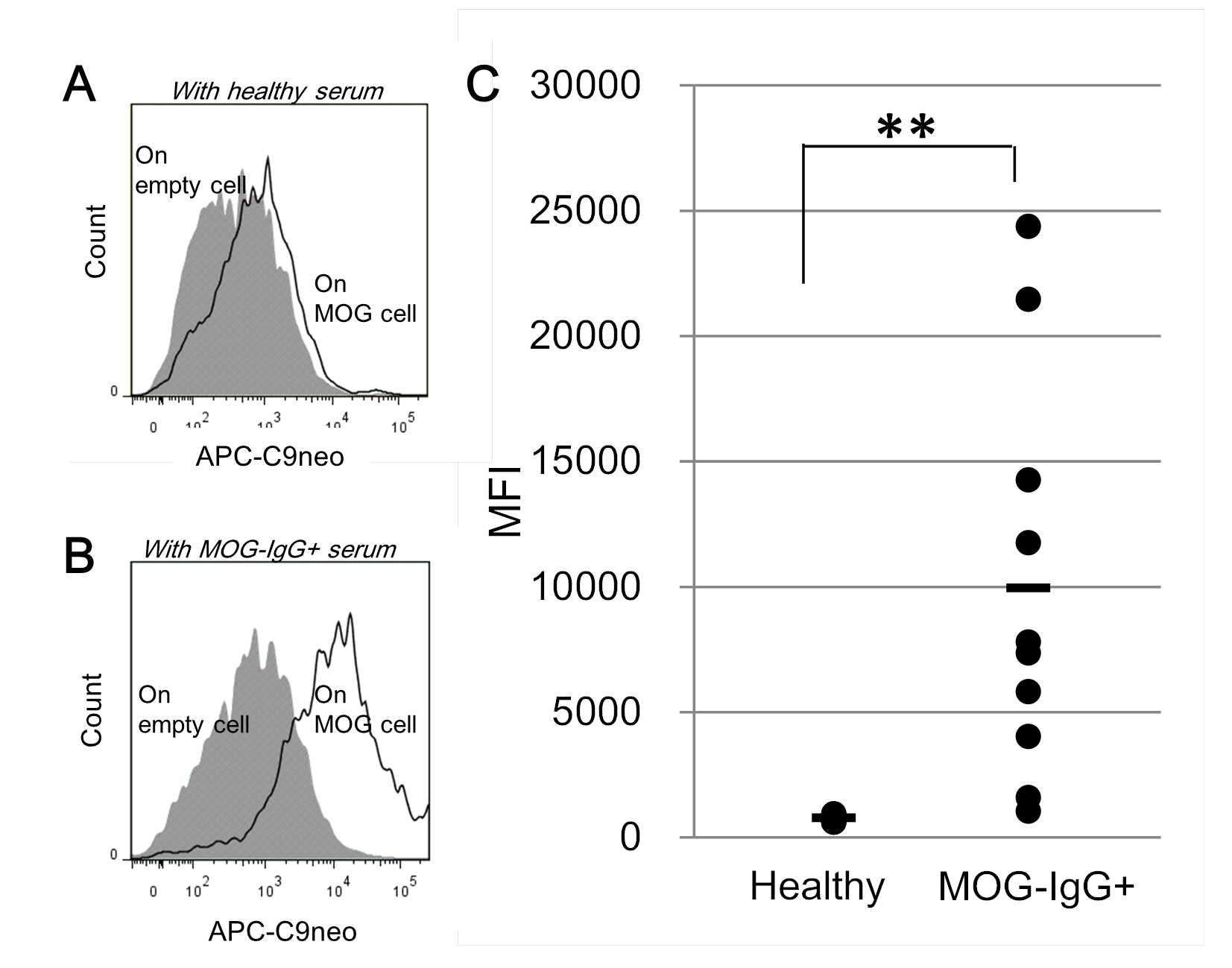

Supplement: Supplementary Figure 6 — Demonstration of membrane attack complex formation by flow cytometry. After the reaction with human sera and the rabbit complement same as cytotoxicity assay, the mixture of MOG-GFP and empty cells is stained with anti-C9neo antibody and detected by flow cytometer. (A,B) Representative histograms of the cells reacted to healthy (A) or MOG-IgG+ (B) serum. (C) Fluorescent level of C9neo was compared between the healthy group and the MOG-IgG positive group. Mann–Whitney U test was used for statistical comparison (**P < 0.01). [file Image_6.TIF]
