# Supplementary material for: Complement-dependent cytotoxicity of human autoantibodies against myelin oligodendrocyte glycoprotein
Source: Front Neurosci. 2023 Feb 1;17:1014071. doi: 10.3389/fnins.2023.1014071 (PMC9930155; doi:10.3389/fnins.2023.1014071)
Supplement: Supplementary Table 1 — Profile of the patients included in the study. ADEM, acute disseminated encephalomyelitis; ON, optic neuritis; MS, multiple sclerosis; NMO, neuromyelitis optica; EM, encephalomyelitis; TM, transverse myelitis; OIND, other inflammatory neurological disease; NIND, non-inflammatory neurological disease. [file Table_1.DOCX]

| Group | Age | Sex | |  | Disease | |
| --- | --- | --- | --- | --- | --- | --- |
|  |  |  |  |  |  |  |
| Patients | 0-16 | Male | 36 |  | ADEM | 12 |
|  | (median; 8) | Female | 36 |  | ADEM+ON | 5 |
|  |  |  |  |  | ON | 5 |
|  |  |  |  |  | MS | 3 |
|  |  |  |  |  | NMO | 2 |
|  |  |  |  |  | EM | 9 |
|  |  |  |  |  | TM | 0 |
|  |  |  |  |  | OIND | 18 |
|  |  |  |  |  | NIND | 18 |
|  |  |  |  |  |  |  |
|  |  | Total | 72 |  | Total | 72 |
|  |  |  |  |  |  |  |
|  |  |  |  |  |  |  |
| Patients | 18-61 | Male | 3 |  | ADEM | 0 |
|  | (median; 33.5) | Female | 11 |  | ADEM+ON | 0 |
|  |  |  |  |  | ON | 4 |
|  |  |  |  |  | MS | 1 |
|  |  |  |  |  | NMO | 1 |
|  |  |  |  |  | EM | 0 |
|  |  |  |  |  | TM | 3 |
|  |  |  |  |  | OIND | 3 |
|  |  |  |  |  | NIND | 2 |
|  |  |  |  |  |  |  |
|  |  | Total | 14 |  | Total | 14 |
|  |  |  |  |  |  |  |
|  |  |  |  |  |  |  |
| Healthy | 26-40 | Male | 5 |  |  |  |
|  | (median; 32) | Female | 6 |  |  |  |
|  |  |  |  |  |  |  |
|  |  | Total | 11 |  |  |  |
|  |  |  |  |  |  |  |
