# Supplementary material for: Complement-dependent cytotoxicity of human autoantibodies against myelin oligodendrocyte glycoprotein
Source: Front Neurosci. 2023 Feb 1;17:1014071. doi: 10.3389/fnins.2023.1014071 (PMC9930155; doi:10.3389/fnins.2023.1014071)
Supplement: Supplementary Table 2 — IgG subclasses of anti-MOG antibodies. MOG-GFP cells are sequentially reacted with sera and IgG subclass-specific secondary antibodies and are analyzed by flow cytometry. The number of patients who are positive for each IgG subclass is shown. ADEM, acute disseminated encephalomyelitis; ON, optic neuritis; NMO, neuromyelitis optica; EM, encephalomyelitis. [file Table_2.DOCX]

| Group | IgG1 | IgG2 | IgG3 | IgG4 | IgG1+IgG3 |
| --- | --- | --- | --- | --- | --- |
|  |  |  |  |  |  |
| ADEM | 5 | 0 | 0 | 0 | 0 |
| ADEM+ON | 3 | 0 | 0 | 0 | 0 |
| ON | 1 | 0 | 0 | 0 | 0 |
| NMO | 1 | 0 | 0 | 0 | 0 |
| EM | 1 | 0 | 0 | 0 | 1 |
|  |  |  |  |  |  |
| Total | 11 | 0 | 0 | 0 | 1 |
